# Smooth muscle actin and s100p on non germinal centre diffuse large B cell lymphoma are adverse prognostic factors: pilot study

**DOI:** 10.1186/1746-1596-2-9

**Published:** 2007-03-02

**Authors:** Howayda Abd El All

**Affiliations:** 1 Department of Pathology, Faculty of Medicine, Suez Canal University, Ismailiya, Egypt; 2 Immunohistochemistry Laboratory, Nasser Institute, MOHP, Cairo, Egypt

## Abstract

**Introduction:**

The expression of smooth muscle actin (SMA) and s100p has been identified on an aggressive retro-orbital diffuse large B cell lymphoma (DLBCL) [1].

**Aim:**

To assess the prognostic significance of immunohistochemical (IHC) expression of SMA and s100p on DLBCL.

**Materials and methods:**

Twenty nine cases diagnosed as DLBCL were first classified into germinal centre (GC) B cell like and non GC origin either activated B cells (ABC) or type 3 based on their immunoreactivity for CD10, bcl-6 and Mum-1. Bcl-2 and MIB-1 as adverse prognostic factors were assessed. SMA and s100p were evaluated and correlated with patients' clinicopathological characteristics.

**Results:**

Eleven cases (37.93%) positive for CD10 and/or bcl-6 were classified as GC B cell like subtype, 7 cases positive only for Mum-1 as ABC subtype (24.14%), and 11 cases double positive or negative for bcl-6 and Mum-1 were considered as type 3 (37.93%). Nuclear and cytoplasmic SMA and s100p were expressed in 58.62% and 51.72% of cases respectively and were strongly associated with the non GC like phenotype (p < 0.001 for SMA and p < 0.01 for s100p). SMA and s100p were strongly related to each other (p < 0.001). SMA was closely associated with bcl-2 and MIB-1 (p < 0.01 and p < 0.025 respectively), while s100p was only associated with bcl-2 (p < 0.05).

**Conclusion:**

SMA and s100p are expressed on non GC DLBCL and appear to be adverse prognostic factors. Future large studies evaluating their significance will be valuable to assess the different subgroups in clinical context. Lastly, this expression may lead to misdiagnosis of non hematopoeitic neoplasm if lymphoid markers are not included in the IHC panel.

## Background

The identification of a DLBCL positive for both SMA and s100p was accidentally discovered during the diagnosis of a retro-orbital mass of 3 months duration in a 37 years old female [1]. Clinical and radiological differential diagnosis was rhabdomyosarcoma, melanoma and lymphoma. An initial panel included s100p, HMB45, SMA and LCA. Surprisingly, s100p, SMA and LCA were positive on the neoplastic cells. Further immunostaining demonstrated positivity of the neoplastic cells for CD20 and CD79a classifying the lesion as DLBCL, centroblastic polymorph variant on histopathological basis. In addition, bcl-2 was positive while CD10, bcl-6 and CD138 were negative indicating that this DLBCL neither of GC nor of ABC phenotype, was type 3 according to recent data of gene profiling [2,3]. Patient died shortly after the diagnosis.

DLBCL as defined by the WHO classification [4] is an umbrella term comprising heterogeneous biological entities at the molecular and clinical levels that cannot be distinguished by morphologic or immunophenotypic analysis [5,6]. However, gene expression profiling divided DLBCL into important subgroups with regard to prognosis as GC B cell like, ABC like and type 3 where the GC B cell like group shows significantly better survival compared to the other two groups [2,3]. Recently, bcl-6, CD10 and Mum-1 have been shown to be differently expressed in the three phenotypes; both CD10 and bcl-6 are considered as GC markers, Mum-1 is expressed in the ABC group while type 3 represents a grey zone negative for CD10 [2,3,7]. Other markers of prognostic significance include bcl-2 [8-10] and MIB-1 [11], both are adverse prognostic factors.

In the present work, we evaluated the expression of SMA and s100p on DLBCL, and correlated this expression with the site of presentation either nodal or extranodal, histologic variants, GC or non GC phenotype and other patient's clinicopathological characteristics. The clinical outcome was assessed whenever possible.

## Materials and methods

### Patients and materials

Twenty nine cases diagnosed as de novo DLBCL either nodal or extranodal, were retrieved from the Immunohistochemistry Laboratory of Nasser Institute. Patients with immunodeficiency-associated or post transplant tumors were excluded. They were classified according to the WHO classification [4] into centroblastic when more than 90% of the cells were centroblasts, centroblastic polymorph when the proportion of immunoblasts ranged from 10% to 90%, and immunoblastic when more than 90% of tumor cells were immunoblasts. The presence of Reed-Sternberg like cells or cells with anaplastic features, plasmacytoid, spindle and clear cells with abundant cytoplasm were reported for each case whenever identified. Patients' characteristics, initial presentation, clinical stage and clinical outcome if possible, were retrieved from the pathological files.

### Immunohistochemistry

IHC was effectuated on 5 μm thick paraffin embedded tissue sections. The antibodies in the study, their sources, clones, heat induced epitope antigen retrieval (HIER) buffer and dilutions are illustrated in table 1. HIER was done by heating the slides in microwave (800 watts) for 15 minutes (3 cycles × 5 minutes).

**Table 1:** Antibodies used in the study

Antibodies	Source	Clone/Antibody	HIER	Dilution	Incubation	Staining interpretation
S100p	DakoCytomation	Rabbit polyclonal	1	1: 400	RT	Nuclear -/+cytoplasmic
SMA	DakoCytomation	1A4/monoclonal	2	1:50	RT	Nuclear -/+cytoplasmic
CD20	DakoCytomation	L26/monoclonal	2	1:200	RT	Membranous
CD79a	DakoCytomation	JCB117/monoclonal	2	1:50	RT	Membranous
CD10	Novocastra	NCL-CD10-270/monoclonal	1	1:20	RT	Membranous
Bcl-6	DakoCytomation	BG-B6p/monoclonal	1	1:20	RT	Nuclear
Mum-1	Santa Cruz	goat polyclonal	2	1:50	37°C	Nuclear, nucleolar
Bcl-2	DakoCytomation	124/monoclonal	2	1:50	RT	Membranous
Ki-67	DakoCytomation	MIB-1/monoclonal	1	1:75	RT	Nuclear, nucleolar

1-Tris-EDTA pH9.0

2-Citrate buffer pH6.0

RT: room temperature

In brief, slides were hydrated in descending grades of alcohol followed by distilled water. Endogenous peroxidase activity was quenched by 0.3% hydrogen peroxide for 5 minutes followed by rinsing in distilled water and three times wash in phosphate buffer saline (PBS) pH 7.4. The antibodies were incubated for 30 minutes and then the slides were rinsed in successive bathes of PBS. The revelation was done by the LSAB-2 detection kit (DakoCytomation) according to the manufacturer's instructions. Finally, diaminobenzidine tetrachloride (DAB) was applied for 5 minutes. Slides were counterstained in Harris haematoxylin (Hx), dehydrated, cleared in xyelene and coverslipped. Reactive nodes and internal control were used as positive control for all antibodies. Slides with omitted antibodies were used as negative control.

### Interpretation of IHC

IHC results were evaluated in semi-quantitative manner summarized in table 2. The intensity of staining was assessed, but was not used to determine positivity because paraffin blocks were obtained from different institutions with variability in tissue fixation, processing and storage conditions, factors that appeared to affect the intensity of staining. The staining interpretation is summarized in table 1.

**Table 2:** Guideline for interpretation of IHC scoring

Scoring	% of positive cells
0	absence of staining up to 5%
+	6–25%
++	26–50%
+++	51–75%
++++	>75%

Cases were subdivided into GC like B cells, ABC or type 3, based on the positivity for CD10, bcl-6 and Mum-1. A case was considered of GC like B cell origin if Mum-1 [12] was negative and CD10 alone or both CD10 and bcl-6 were positive [13,14]. ABC was considered when cells were only positive for Mum-1 and type 3 when a case was negative for CD10 but double positive or negative for both bcl-6 and Mum-1.

### Statistical analysis

Chi-square test was used to test the association between SMA and s100p IHC expression on one hand and the subtypes, GC versus ABC versus type 3, and other categorical variables on the other hand. On a latter step, both ABC and type 3 was grouped together as non GC phenotype since type 3 group has similar outcome as ABC group [3]. A cut-off point of 50% was used for MIB-1. The test was considered positive when p value was equal to or less than 0.05.

## Results

Sixteen patients were males and thirteen females. The age ranged from 9–74 years with an average 45.20 years. Eleven cases (37.93%) were negative for Mum-1, but positive for CD10 and/or bcl-6 and were classified as GC B like cells. Seven cases (24.14 %) were CD10-bcl-6-Mum-1+, favouring an ABC subtype. Eleven cases (37.93%) were CD10-bcl-6+Mum-1+ (8/11) or CD10-bcl-6- Mum-1- (3/11) and were considered type 3 (table 3). Case 13 and 27 died shortly after the diagnosis from disseminated disease while the other cases are new ones actually receiving their treatment.

**Table 3:** Clinico-pathological characteristics of the studies cases

N°	Sex/age	stage	Site of biopsy	Histologic variant	CD10	Bcl-6	Mum-1	Bcl-2	MIB-1	subtype
1	F/60	I	CNS, parietal	CBp	-	++	+++	++++	25%	Type 3
2	M/40	I	CNS, extradural	CBp, anaplastic cells	-	-	+++	-	50%	ABC
3	F/55	I	CNS, frontal	CBp	-	++	++++	++++	25%	Type 3
4	F/45	III	Nodal, axillary	T/HRTCL	-	-	+++	++++	90%	ABC
5	M/70	III	Nodal, cervical	CBp	-	++	+++	+++	25%	Type 3
6	F/66	III	Nodal, inguinal	CBp	-	-	+++	++++	75%	ABC
7	M/23	II	Nodal, cervical	CB	+++	++	-	-	50%	GC
8	F/74	I	Nodal, abdominal	CBp	+++	++	-	-	25%	GC
9	M/50	I	Nodal, cervical	CB	-	++	-	-	25%	GC
10	F/52	III	Nodal, inguinal	CBp, anaplastic cells	-	+++	+++	+++	90%	Type 3
11	M/43	III	Nodal, inguinal	CBp	-	-	-	++	90%	Type 3
12	M/55	IV	Nodal, abdominal	CBp, anaplastic cells	-	-	+	++++	75%	ABC
13*	M/34	IV	Nodal, abdominal	CBp, anaplastic, clear cells	-	++	+++	++++	90%	Type 3
14	M/48	III	Nodal, cervical	CBp	-	+++	++	++++	75%	Type 3
15	M/49	I	Nodal, cervical	CB	++	-	-	-	50%	GC
16	F/11	II	Nodal, abdominal	CBp, clear cells	-	-	+	++++	50%	ABC
17	F/9	I	Nodal, cervical	CBp, anaplastic, clear cells	+++	++	-	-	10%	GC
18	M/37	I	Nodal, cervical	CB p, anaplastic cells	+++	+++	-	-	50%	GC
19	M/56	III	Nodal, cervical	CBp, clear cells	-	-	+++	++	90%	ABC
20	F/63	III	Nodal, abdominal	CBp, anaplastic cells	+++	+++	-	-	50%	GC
21	M/31	I	Extranodal, Appendicular	CBp	-	-	++++	-	90%	ABC
22	M/15	I	Extranodal, gastric	CBp	++	++	-	-	90%	GC
23	F/49	I	Extranodal, gastric	CB	-	-	-	-	25%	Type 3
24	M/70	I	Extranodal, gastric	CBp	-	+++	+++	++++	90%	Type 3
25	M/35	I	Extranodal, omentum	CBp	+++	-	-	-	10%	GC
26	M/52	I	Extranodal, spleen	Histiocyte-like, anaplastic cells	-	++	-	-	25%	GC
27*	F/43	I	Extranodal, retro-orbital	CBp	-	-	-	+++	90%	Type 3
28	F/45	I	Extranodal, scapular	CBp, spindle, clear cells	-	++	+++	++++	75%	Type 3
29	F/46	I	Extranodal, iliac bone	CBp, clear cells	+++	++	-	-	10%	GC

CB: centroblastic

CB p: centroblastic polymporph

T/HRTCL: Tcell/histiocyte rich B cell lymphoma

CNS: central nervous system

* dead from the disease

**Figure 1:**
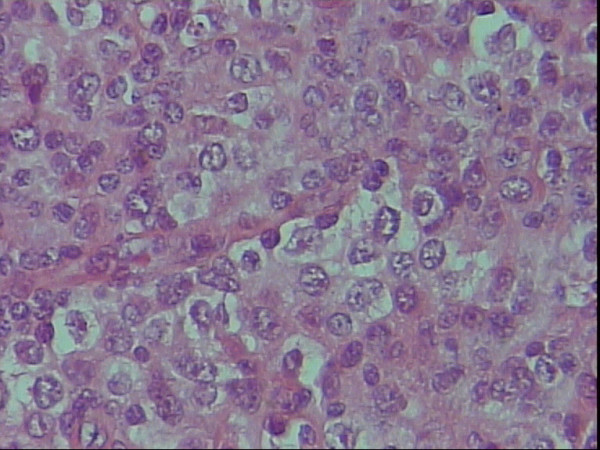
Centroblastic polymorph variant on H&E staining, × 40.

SMA and s100p were expressed in 58.62% and 51.72% of cases respectively and were strongly associated with ABC and type 3 (p < 0.01 for SMA and p < 0.025 for s100p). Figures 1 through 10 illustrate a case of DLBCL, ABC subtype. However, when grouped into GC and non GC subtypes, the association become more strong (p < 0.001 for SMA p < 0.01 for s100p). Noteworthy, both SMA and s100p were strongly related to each other (p < 0.001). In addition, bcl-2 and MIB-1 were significantly expressed in the non GC phenotype whether separated as ABC and type 3 or grouped as non GC group (p < 0.001 for bcl-2 and p < 0.01 for MIB-1) (table 4). SMA was closely associated with bcl-2 and MIB-1 (p < 0.01 and p < 0.025 respectively), while s100p was only associated with bcl-2 (p < 0.05). There was no relationship between the IHC expression of SMA and s100p and age, sex, clinical stage, site of presentation, histological variants and predominant cell type.

**Figure 2:**
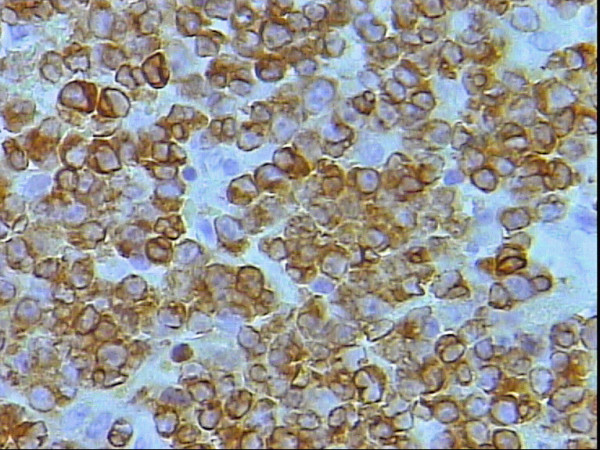
CD79a membranous staining of the lymphocytes, DAB, Hx, × 40.

**Figure 3:**
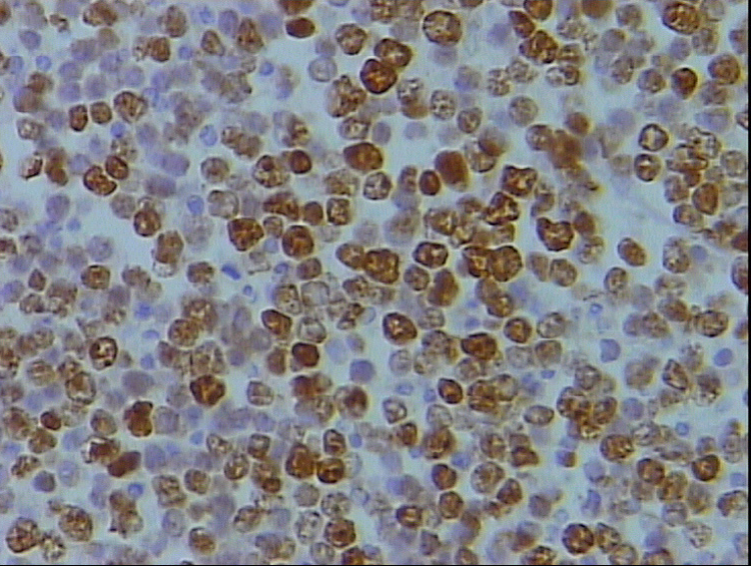
Strong nuclear, nucleolar Mum-1 staining of 50% of the neoplastic lymphocytes, DAB, Hx, × 40.

**Figure 4:**
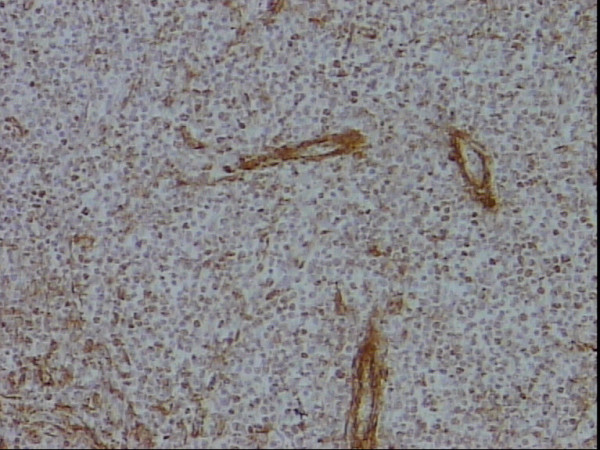
SMA staining showing positivity of the lymphocytes and internal control of the blood vessels, DAB, Hx, × 10.

**Figure 5:**
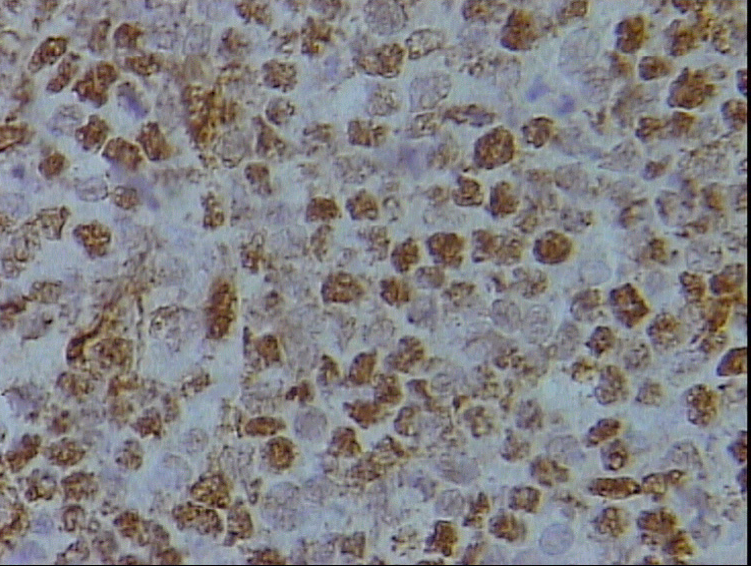
Higher power magnification of figure 4 showing moderate nuclear and rare cytoplasmic SMA staining of the neoplastic lymphocytes, DAB, Hx, × 40.

**Figure 6:**
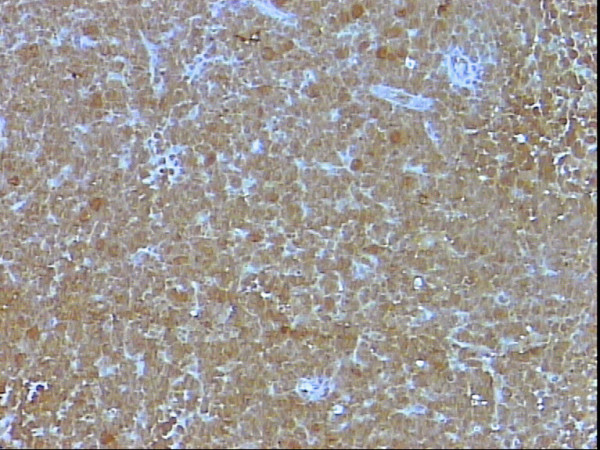
Nuclear staining of s100p of all neoplastic lymphocytes, DAB, Hx, × 10.

**Figure 7:**
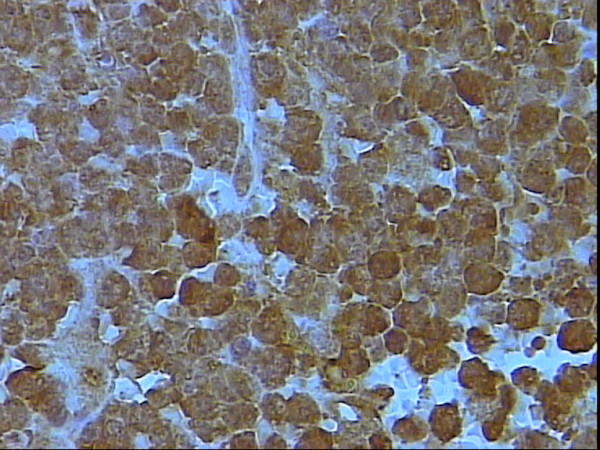
Higher power magnification of figure 6 showing moderate to strong nuclear for s100p, DAB, Hx, × 40.

**Figure 8:**
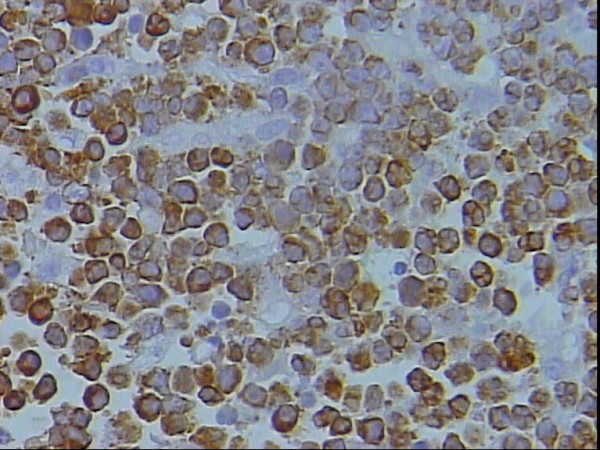
Bcl-2 membranous staining of the neoplastic lymphocytes, DAB, Hx, × 40.

**Figure 9:**
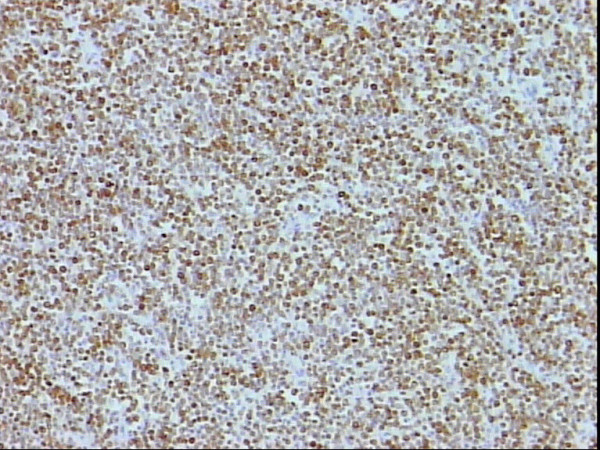
High proliferation fraction as noted by the staining of up to 75% of the neoplastic lymphocytes, MIB-1, DAB, Hx, × 10.

**Figure 10:**
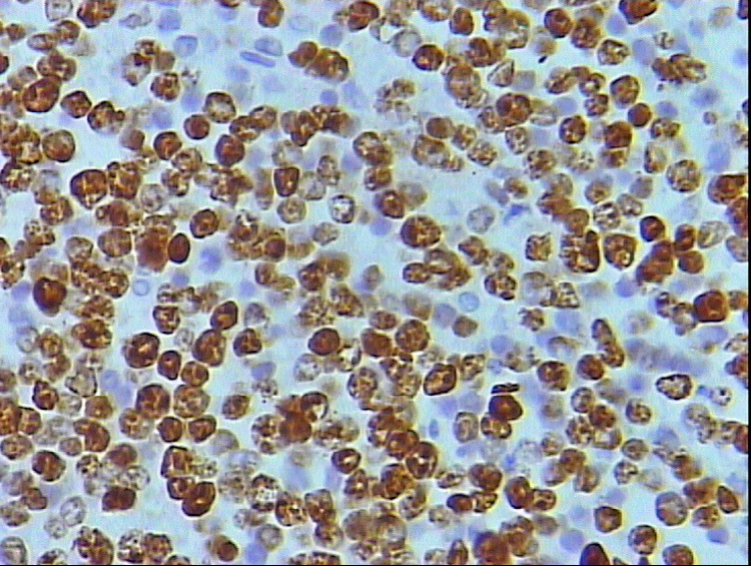
Higher power magnification of figure 9 showing strong nuclear staining of the neoplastic lymphocytes, MIB-1, DAB, Hx, × 40.

**Table 4:** correlation between DLBCL subgroups and SMA, s100p, bcl-2 and MIB-1

Subtype	s100p (p = 0.025)					SMA (p < 0.01)				bcl-2 (p < 0.001)				MIB-1 (p < 0.01)		Total
		
	-	+	++	+++	++++	-	++	+++	++++	-	++	+++	++++	<50%	>50%	
GC	9	2	0	0	0	10	1	0	0	11	0	0	0	10	1	11
ABC	2	0	1	1	3	0	2	1	4	2	1	0	4	2	5	7
type 3	3	0	4	2	2	2	5	2	2	1	1	3	6	4	7	11
Total	14	2	5	3	5	12	8	3	6	14	2	3	10	16	13	29

## Discussion

SMA detects actin in smooth muscle, while s100 p is present in glial cells, schwann cells, satellite cells, fat cells, skeletal and heart muscle cells, melanocytes, chondrocytes, myoepithelial cells, some glandular epithelia and follicular dendritic cells. In the English literature, few reports identified SMA on B lymphocytes and B cell lymphoma (BCL), while s100p was observed on T cells and T cell lymphoma (TCL). However, both markers have never been reported together on BCL. Actin was thought to be present on the surface of B and to a lesser extent on T cells; an initial report with immunofluorescence misinterpreted as a misleading result reflecting the presence of antibodies cross reacting with immunoglobulin [15]. Further studies identified actin as a major protein of human lymphocytes [16-19].

Although this pilot study involved a limited number of cases, it is the first report to our knowledge evaluating the immunoreactivity of both SMA and s100p on DLBCL and associating this expression with the GC B like and non GC phenotype either ABC or type 3. We identified SMA and s100p on non GC DLBCL phenotype pointing out to the adverse effect of both markers, as recent data showed that the non GC phenotype is a poor risk group [2,3]. Moreover, both markers were associated with bcl-2 expression, a known adverse prognostic factor in DLBCL [8-10]. However, only SMA had a strong relationship with high proliferation fraction as assessed by MIB-1 immunostaining. This point is concordant with earlier research showing that human B cell activation by receptor-mediated stimuli, results in actin polymerization changes in the cytoskeleton, messages transduction and proliferation in B lymphocytes [20]. Moreover, it has been proposed that gelsolin, an actin-regulatory protein that modulates actin assembly and disassembly promote cell growth through inhibiting the apoptotic cell death program by a mechanism independent from the bcl-2 family [21]. Does this mechanism operate also on lymphoma cells? An interesting point that needs to be answered. Furthermore, the nuclear expression of actin in the present work, adds more evidence on the aggressive nature of SMA+DLBCL as this expression has an important role in nucleosome remodelling structure, transcription, and cell growth [22]. Other studies implicated that nuclear actin interacts with RNA polymerase II and may have function on the RNA polymerase II-mediated transcription [23,24]. The clinical impact of such immunoreactivity was evaluated in two patients, classified as non GC phenotype (cases 13 and 27), strongly expressing SMA, s100p, bcl-2 and a high proliferation fraction. Unfortunately, those patients died shortly after diagnosis.

The expression of SMA on DLBCL has been published in two reports. A 64-year-old male patient presented with disseminated large non-cleaved BCL that exhibited sarcomatoid and myxoid patterns and was strongly positive for SMA. However, despite vigorous chemotherapy, the patient died 3 months later [25]. The second report documented that four out of five extranodal DLBCL presenting with prominent spindle cell morphology were actin positive [26]. However, we failed to found an association between SMA immunoreactivity and histologic variants and cellular morphology as sarcomatoid, anaplastic, clear cell or spindle cells. In non haematological malignancies, it has been shown that the multidrug resistant osteosarcoma cells exhibited a remarkable increase in well-organized actin stress fibres; furthermore, dibydrocytoebalasin B, a specific inhibitor of actin polymerization, that dramatically disrupted this network of stress fibres, increased the intracellular accumulation of doxorubicin (DOX) and modified the resistance against DOX [27].

To our knowledge, the expression of s100p on B cells or BCL has never been reported in the English literature while it has been described on T cells expressing CD8 or CD4. One of the earliest reports documented that s100p+ T lymphocytes were CD8+ small lymphocytes with poorly developed cellular organelles and unclear function [28]. The expression of s100p on T cell lymphoproliferative disorders has been associated with poor clinical outcome for all the reported cases. Initially, a report identifying a tumor with intermediate features between TCL and malignant histiocytosis has been described; patient had fever, pancytopenia with relative increase of CD8 lymphocytes, severe bone marrow (BM) hypoplasia, generalized lymphadenopathy and splenomegaly [29]. A rapidly fatal disease of a 12 years old boy presenting with sinusoidal pattern of paraoaortic nodal involvement, thrombocytopenia, splenomegaly and BM involvement was s100p+ CD4+ CD8- TCL [30]. Three out of four cases of s100+ alpha/beta chain TCL presenting with hepatosplenomegaly and CNS involvement had a fatal outcome despite treatment [31]. A second case of s100p+ hepatosplenic alpha/beta TCL and pancytopenia has been described [32]. A 12 years old boy died shortly after diagnosis from an aggressive s100p+ CD56+ non-nasal T cell lymphoproliferative disorder presenting with splenic and marrow involvement [33]. In non haematological malignancies, s100p expression was significantly associated with decreased survival and was an independent predictor of poor prognosis in non-small cell lung cancer [34].

In conclusion, this is the first observation to report the expression of both SMA and s100p on non GC DLBCL. These markers appear to be adverse prognostic factors. Future studies evaluating the significance of these markers in conjunction with other biomarkers will be valuable to assess the different subgroups in clinical context. Lastly, the observed SMA and s100p reactivity in DLBCL may lead to a misdiagnosis of non-haematopoietic malignancies if lymphoid markers are not included in the IHC panel.

## Abbreviations

DLBCL: diffuse large B cell lymphoma, germinal centre: GC, activated B cells: ABC, SMA: smooth muscle actin, CNS: central nervous system, CB: centroblastic, CB p: centroblastic polymporph, BCL: B cell lymphoma, TCL: T cell lymphoma.

## Competing interests

The author(s) declare that they have no competing interests.
